# Total hip arthroplasty (THA) in a patient with subcapital femoral neck fracture and ipsilateral above-knee amputation: A case report

**DOI:** 10.1016/j.tcr.2024.101027

**Published:** 2024-04-08

**Authors:** Francesco Maria Milella, Massimiliano Paolocci, Emanuele Persi, Riccardo Mezzoprete

**Affiliations:** a Azienda Ospedaliera San Camillo de Lellis – Orthopaedics and Traumatology department, Viale J.F. Kennedy, 02100 Rieti, Italy; b Orthopaedics and Traumatology Division - Department of Anatomical, Histological, Forensic Medicine and Orthopaedics Sciences, University “La Sapienza”, Piazzale Aldo Moro 5, 00185 Rome, Italy

**Keywords:** THA, Femoral neck fracture, Above-knee amputation, Subcapital fracture in amputee patient

## Abstract

This case report describes the surgical and post-operative challenges encountered following a THA performed for a subcapital femoral neck fracture in a patient with an ipsilateral above-knee amputation.

## Introduction

Femoral neck fractures in amputee patients pose a challenge for orthopaedic surgeons and the entire team involved in post-operative rehabilitation. These fractures are uncommon, and the literature on the topic is limited. [[1], [2], [3], [4], [5], [6], [7], [8], [9]]

Management of femoral neck fractures in patients with above-knee amputations is particularly challenging for the surgeon. [[6], [7], [8], [9]] This is primarily due to the difficulties involved in the intraoperative management of the femoral stump. The altered anatomy and disuse osteoporosis typical of these patients can lead to diagnostic errors, favor immobilization, make surgical reduction and stabilization more difficult, and ultimately slow down post-operative rehabilitation. [[5], [6], [7], [8], [9], [10]]

In a patient with a femoral neck fracture who uses an external prosthesis to ambulate, it is essential to prioritize a return to pre-injury activities as quickly as possible.

This study presents the case of a patient with a right above-knee amputation who suffered a type 31B1.3 AO subcapital fracture on the same side, which was treated with a total hip replacement.

## Case report

An 85-year-old male patient presented to the emergency department of our hospital in July 2023 with a low-impact trauma and right hip pain following a fall from the toilet. He was in a septic state with fever, anuria, and asthenia. The patient's past medical history included moderate-grade cardiopathy, COPD, epilepsy, and an indwelling urinary catheter. In 2021, he underwent an above-knee amputation of the right femur due to obliterating vasculopathy of the lower limbs.

**Fig. 1:**
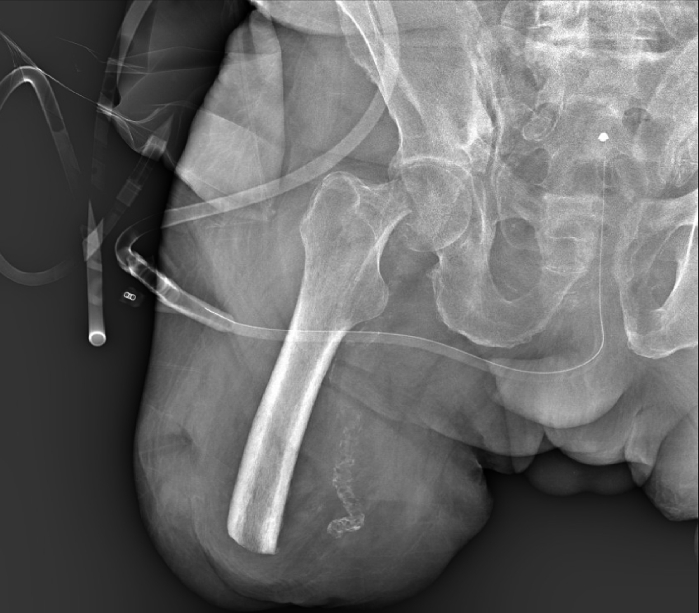
Subcapitate fracture AO/OTA 31B1.3.

**Fig. 2:**
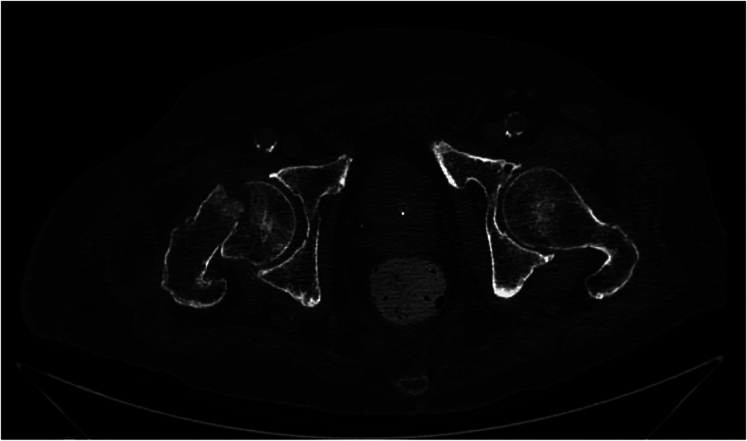
Subcapitate fracture axial CT showing decomposition.

At the physical examination the trochanteric and inguinal regions were tender and painful. The overlying skin was intact, with a well-healed surgical scar at the distal end of the stump, consistent with an above-knee amputation. No peripheral vascular or neurological deficits were apparent. No other injuries were identified. Radiographs showed a type 31B1.3 AO subcapital fracture (Fig. 1, Fig. 2).

Due to septic shock originating from the indwelling urinary catheter, the patient was admitted to the internal medicine department of our hospital for treatment.

In September 2023, after hemodynamic stabilization, approximately two months after his admission to the emergency department, the patient underwent a pre-operative orthopaedic consultation and was scheduled for a THA. According to a previous infectious disease video consultation, the patient was colonized in the lower urinary tract with *Pseudomonas Aeruginosa and Klebsiella pneumoniae* mdr despite antibiotic therapy. Therefore, in addition to the usual pre-operative antibiotic prophylaxis used in our center (cefazolin 2 g IV 30 min before surgery), 1 vial of ceftazidime/avibactam 2 g/0.5 g was administered on the evening before surgery and repeated for two days post-operatively, as prescribed by the infectious disease specialist.

A THA was decided upon due to the patient's high functional demand. Prior to the trauma, and despite his numerous comorbidities, the patient was active and regularly exercised with his external prosthesis.

After obtaining informed consent, the procedure was performed under subarachnoid anesthesia, with the patient in the left lateral decubitus position and using a direct lateral approach to the right hip approximately 20 cm long. This was to facilitate the management of the femoral stump during the dislocation maneuvers. No Steinmann pins or other instruments were inserted in anatomic landmarks to facilitate the control of the stump. A 48 mm porous coated uncemented acetabular shell (Adler Ortho) was aligned with the transverse acetabular ligament. A cementless size 4 type femoral stem was inserted (Adler Ortho) and a 28 mm hooded Polyethylene liner was used (Adler Ortho). After trials, a 28 mm femoral head alumina ceramic (Adler Ortho) was impacted and the hip was reduced.

Accurate attention was paid to correctly aligning the femoral stem, respecting its anteversion.

The preparation of the femoral canal was possible thanks to the use of a Verbrugge bone-holding forceps, which was used to gently extrarotate the femoral stump.

Since the patella could not be used as a reference point, it was decided to orientate ourselves by palpating the lesser trochanter and, visually, on the orientation of the femoral canal at the level of the femoral neck osteotomy.

**Fig. 3:**
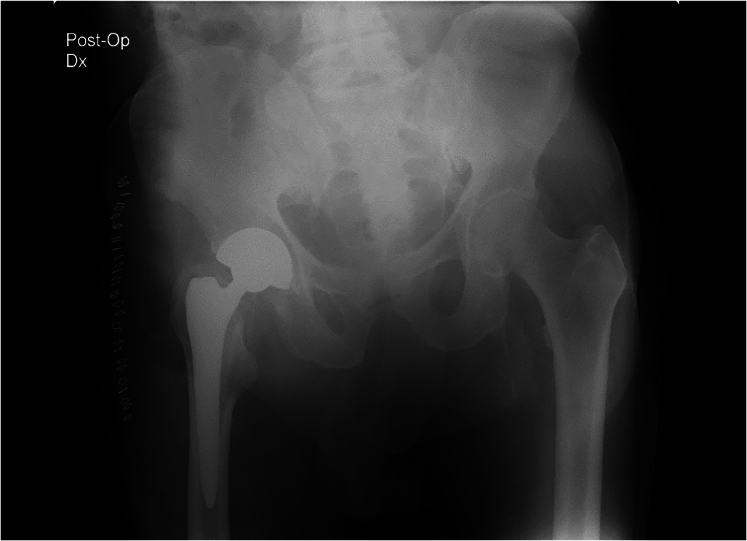
RX post-operative control.

**Fig. 4:**
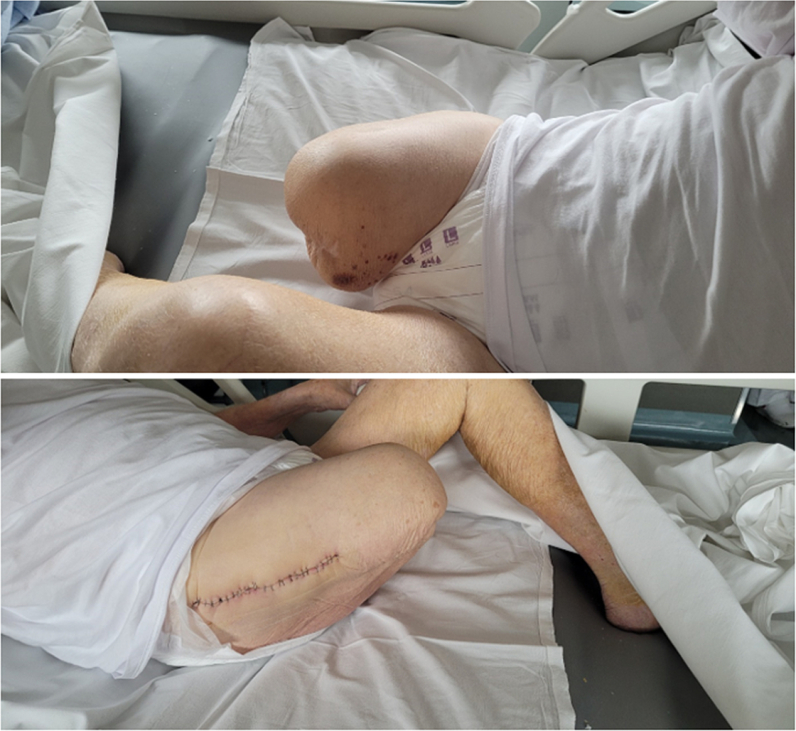
Post-operative skin condition.

**Fig. 5:**
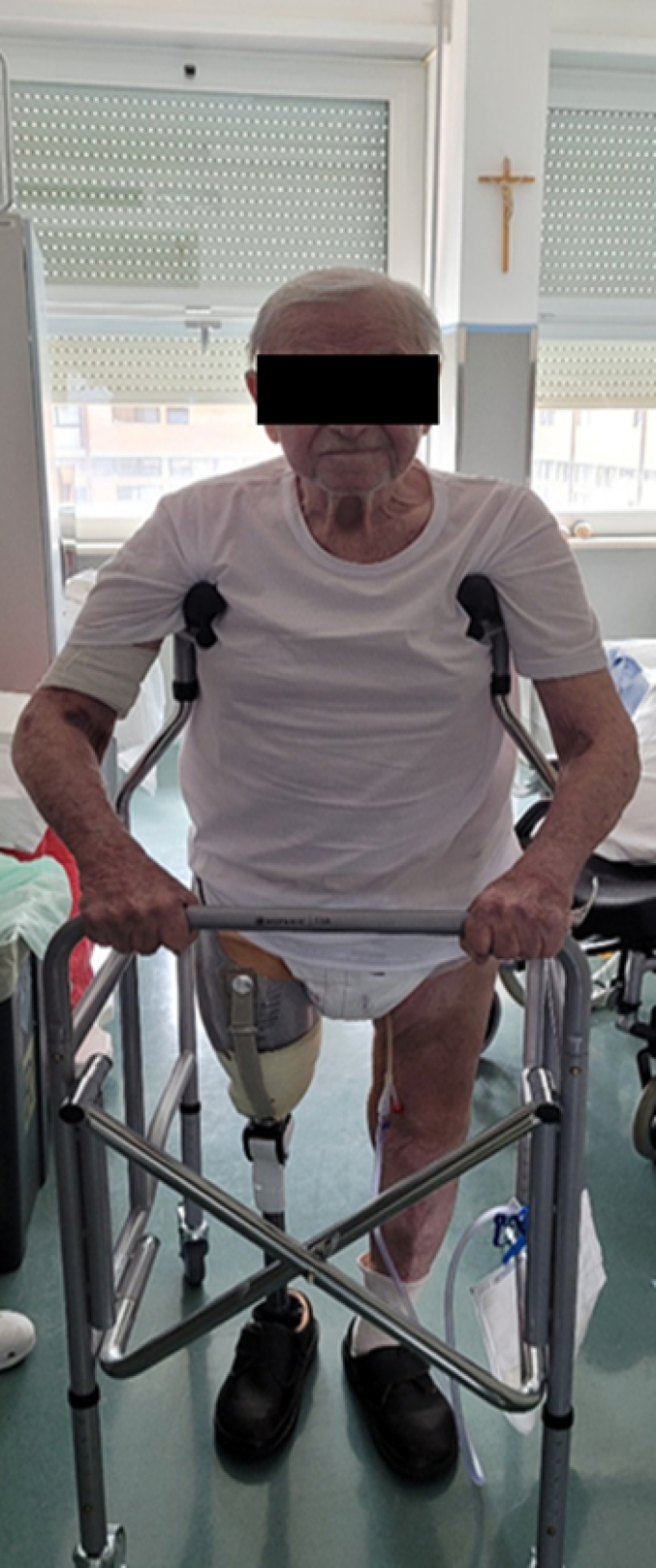
The patient takes the first steps.

The patient received post-operative antibiotics and anticoagulant therapy, according to departmental protocols. No complications occurred in the post-operative period (Fig. 3, Fig. 4). In the immediate post-operative period, the patient began appropriate physiotherapy: Strengthening of the lower limb muscles, readaptation of the stump to the external prosthesis, progressive re-education of the gait pattern. After two weeks, the patient was able to stand and began to walk with the help of a physiotherapist and appropriate aids (Fig. 5). Three months after the trauma, he was able to walk without aids for short distances. During the first month, the use of the prosthesis was uncomfortable due to significant swelling of the stump, which subsided gradually with the use of an elastic bandage.

**Fig. 6:**
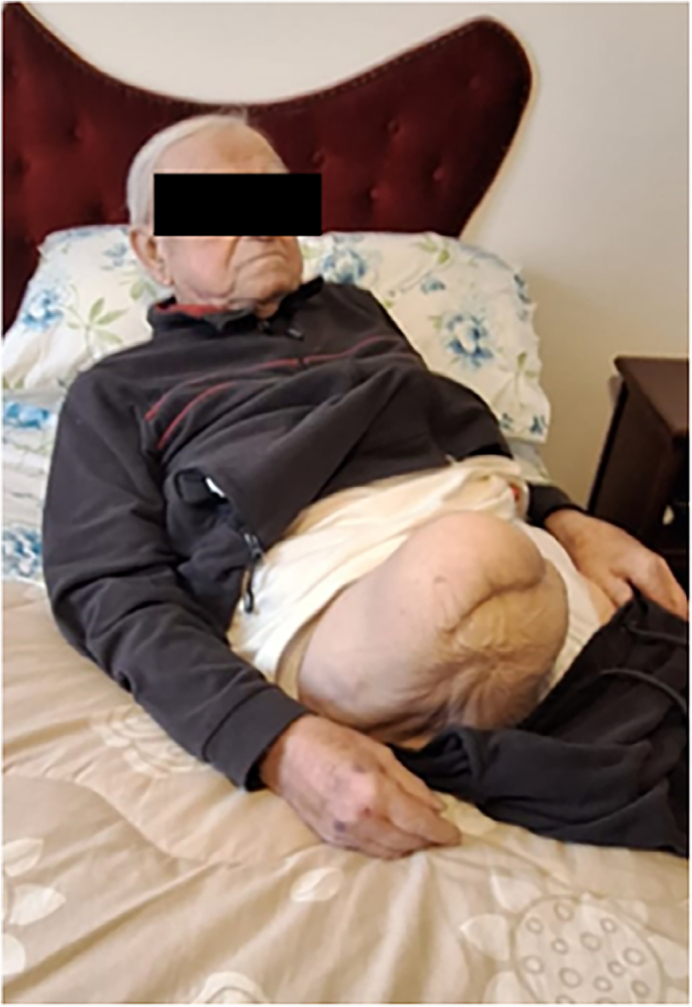
The patient 6 months after surgery.

**Fig. 7:**
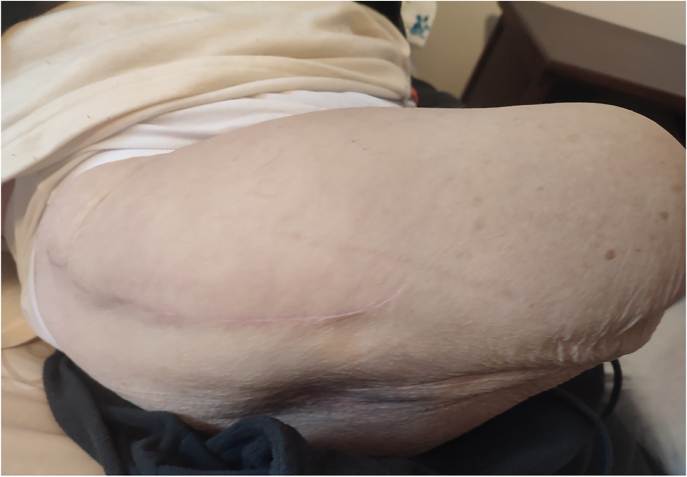
Close-up of the surgical wound 6 months after surgery.

At the 6-month follow-up, the amputated limb was in good condition (Fig. 6, Fig. 7). On objective examination, the hip joint was completely mobile, with no pain. No leg length discrepancy was noted when wearing the prosthesis. Radiographs showed well-incorporated femoral and acetabular components without any sign of osteolysis.

## Discussion

We have reported a case of subcapital femoral fracture in a patient with above-the-knee amputation. The surgical challenge arises from the reduced lever arm due to the femoral stump, the absence of the patellar landmark to guide the correct anteversion of the femoral stem, and the increased delicacy required in handling the osteoporotic stump [10,11]. Despite the waiting period for the Total Hip Arthroplasty (THA) procedure due to the concurrent septic shock condition, the patient showed no clinical or radiographic improvement. Conservative treatment of a femur with a short lever arm may be considered, provided there are no displaced cervical or intertrochanteric fractures [12,13]. In our specific case, the severe displacement, pre-existing coxarthrosis, and respect for the patient's functional expectations necessitated a THA surgical treatment. Utmost attention was paid to the surgical access, which, although extensive to facilitate dislocation maneuvers, was extended in such a manner to avoid creating conflict or pressure on the external prosthesis. Postoperatively, physical therapists paid considerable attention to the readjustment of the stump on the external prosthesis. The post-operative edema, associated with that due to disuse from the fractured limb, had caused an increase in the diameters of the stump, consequently making it difficult to comfortably wear the external prosthesis. The use of elastic bandages applied in a manner to achieve gradual compression that stimulated venous return, allowed the readjustment of the stump to the external prosthesis in less than two weeks. Given the results achieved, following careful preoperative planning and considering the patient's functional expectations, we believe it is always appropriate to evaluate surgery in ambulatory above-the-knee amputees, so as to ensure a rapid return to daily activities and social life.

## Funding

No grant has been received for this study.

## Ethical approval

Not required.

## Informed consent

All the patients gave their approval via informed consent to publish their clinical and laboratory data, within utterly lawful respect of privacy.

## CRediT authorship contribution statement

**Francesco Maria Milella:** Conceptualization, Data curation, Formal analysis, Funding acquisition, Investigation, Methodology, Project administration, Resources, Software, Visualization, Writing – original draft, Writing – review & editing. **Massimiliano Paolocci:** Supervision. **Emanuele Persi:** Supervision. **Riccardo Mezzoprete:** Supervision, Validation.

## Declaration of competing interest

None.

## References

[1] Mirdad T., Khan M.R.H., Kazarah Y. (1997). Fracture of the femur in amputation stumps. Ann. Saudi Med..

[2] Boussakri H., Alassaf I., Hamoudi S. (2015). Hip arthroplasty in a patient with transfemoral amputation: a new tip. Case Rep. Orthop..

[3] Perumal R, Gaddam SR, Vasudeva J, Dheenadhayalan J, Rajasekaran S. Bipolar hemiarthroplasty in a patient with above-knee amputation: surgical technique. J. Orthop. Case Rep. 2017;7(1):54–57. doi: 10.13107/jocr.2250-0685.686. [PMC free article] [PubMed] [CrossRef] [Google Scholar]. 10.13107/jocr.2250-0685.686 PMC5458699 28630841

[4] Patnaik S, Nayak B, Sahoo AK, Sahu NK. Minimally invasive total hip replacement in an ipsilateral post-traumatic above-knee amputation: a case report. J. Orthop. Case Rep. 2017;7(2):3–6. doi: 10.13107/jocr.2250-0685.722. [PMC free article] [PubMed] [CrossRef] [Google Scholar]. 10.13107/jocr.2250-0685.722 PMC5553831 28819590

[5] Ma C., Lv Q., Yi C., Ma J., Zhu L. (2015). Ipsilateral total hip arthroplasty in patient with an above-knee amputee for femoral neck fracture: a case report. *Int*. J. Clin. Exp. Med..

[6] Kandel L, Hernandez M, Safran O, Schwartz I, Liebergall M, Mattan Y. Bipolar hip hemiarthroplasty in a patient with an above knee amputation: a case report. J. Orthop. Surg. Res. 2009;4:30. doi: 10.1186/1749-799X-4-30. [PMC free article] [PubMed] [CrossRef] [Google Scholar]. PMC2734558 19646230

[7] Huguet J, Mariscal G, Balfagón A, Mayorga D, Ulldemolins P, Guillot A, Barrés M. Management and outcomes of hip fractures in lower limb amputees: a case series. Indian J. Orthop. 2023 Apr 11;57(7):1063–1067. doi: 10.1007/s43465-023-00890-x. PMID: 37384017; PMCID: PMC10293151. PMC10293151 37384017

[8] Gruhonjic I, West D. Management of femoral neck fracture in above knee amputee with femoral neck system: a case report. J. Orthop. Case Rep. 2022 Aug;12(8):5–8. doi: 10.13107/jocr.2022.v12.i08.2942. PMID: 36687489; PMCID: PMC9831227. 10.13107/jocr.2022.v12.i08.2942 PMC9831227 36687489

[9] Christidis P, Kantas T, Kalitsis C, Frechat SG, Biniaris G, Gougoulias N. Total hip arthroplasty for an intracapsular femoral neck fracture of high-femoral amputee. Arch. Clin. Cases 2022 Jul 7;9(2):50–55. doi: 10.22551/2022.35.0902.10203. PMID: 35813498; PMCID: PMC9262082. 10.22551/2022.35.0902.10203 PMC9262082 35813498

[10] Haleem S, Yousaf S, Hamid T, Nagappa S, Parker MJ. Characteristics and outcomes of hip fractures in lower limb amputees. Injury 2021 Apr;52(4):914–917. doi: 10.1016/j.injury.2020.10.017. Epub 2020 Oct 5. PMID: 33041015. 33041015

[11] Kulkarni J., Adams J., Thomas E., Silman A. (1998). Association between amputation, arthritis and osteopenia in British male war veterans with major lower limb amputations. Clin. Rehabil..

[12] Denton J.R., McClelland S.J. (1985). Stump fractures in lower extremity amputees. J. Trauma.

[13] Bowker J.H., Rills B.M., Ledbetter C.A., Hunter G.A., Holliday P. (1981). Fractures in lower limbs with prior amputation. A study of ninety cases. J. Bone Joint Surg. Am..

